# Gaining insights into Alzheimer’s disease by predicting chromatin spatial organization

**DOI:** 10.1093/bioadv/vbaf268

**Published:** 2025-10-25

**Authors:** Camilo Villaman, Irene Cartas-Espinel, Mauricio Saez, Alberto J M Martin

**Affiliations:** Laboratorio de Redes Biológicas, Fundación Ciencia & Vida, Avenida del Valle Norte 725, Huechuraba, Santiago, 8580702, Chile; Laboratorio de Investigación en Salud de Precisión, Departamento de Procesos Diagnósticos y Evaluación, Facultad de Ciencias de la Salud, Universidad Católica de Temuco, Manuel Montt 56, Temuco, 4813302, Chile; Centro de Investigación, Innovación y Creación UCT, Universidad Católica de Temuco, Rudecindo Ortega 03694, Temuco, 4781509, Chile; Laboratorio de Investigación en Salud de Precisión, Departamento de Procesos Diagnósticos y Evaluación, Facultad de Ciencias de la Salud, Universidad Católica de Temuco, Manuel Montt 56, Temuco, 4813302, Chile; Laboratorio de Redes Biológicas, Fundación Ciencia & Vida, Avenida del Valle Norte 725, Huechuraba, Santiago, 8580702, Chile; Escuela de Ingeniería, Facultad de Ingeniería, Universidad San Sebastián, Bellavista 7, Recoleta, Santiago, 8420524, Chile

## Abstract

**Motivation:**

CTCF is a conserved protein involved in the establishment and maintenance of topologically associating domains (TADs) and loops. Alzheimer’s disease (AD) represents the most common form of dementia, affecting over 50 million elderly individuals. Epigenetic alterations are a hallmark of AD, and epigenetic disruptions are able to affect CTCF binding and looping. Understanding the dynamics of CTCF loops behind AD may lead to new, undiscovered contributions of CTCF to the etiology of AD. To understand the dynamics behind CTCF loops, we developed a CTCF loop predictor using different genomic and epigenomic features, such as CTCF motif information, CTCF protein binding information, and different histone marks.

**Results:**

We obtained F-scores of over 0.9 in GM12878 and K562 cell lines. We reported the importance of each feature in classification, and compared the results with other loop predictors. After testing the predictor, we predicted loops in control and AD data, reported a score of loop disruption and selected the top disrupted loops on AD which were all previously linked with AD in bibliography. Our study contributes to a better understanding of the role of CTCF binding and CTCF loops in gene regulation, and highlights new clues about CTCF in the etiology and development of AD.

**Availability and implementation:**

The method can be found in https://github.com/networkbiolab/jalpy.

## 1 Introduction

CTCF (CCCTC-binding factor) is a conserved zinc finger protein capable of DNA binding (Kim *et al.* 2015) involved on multiple different biological processes. CTCF was first described as a negative regulator of the myc gene in chicken (Klenova *et al.* 1993), and later it was described as a transcriptional regulator involved on many cellular events such as insulation (Jia *et al.* 2020), alternative splicing (Alharbi *et al.* 2021), and loop formation (Xi and Beer 2021). CTCF is reported as the most important insulator in mammals (Kim *et al.* 2007), and is capable of blocking interactions between enhancers and promoters. CTCF is involved on the establishment and maintenance of topologically associated domains (TADs) (Nanni *et al.* 2020), which are domains of increased self-interaction involved with gene regulation. CTCF is enriched at TAD boundaries and blocks interactions from elements inside the TAD with elements outside it (Jia *et al.* 2020).

CTCF is involved with tridimensional genome organization as it can interact with different proteins forming tridimensional functional domains called CTCF loops (Zhang *et al.* 2023). The mechanism behind CTCF loop formation is not completely described, but the loop extrusion model states that cohesin loads into DNA and extrudes the DNA through a cohesin ring, moving along the DNA until reaching bound CTCF anchors, which can form CTCF homodimers interacting with the cohesin ring at the base of the loop (Hansen 2020). Since CTCF and CTCF loops are involved in maintaining domain boundaries or blocking enhancer activities, CTCF binding is critical in regulation of cell gene expression (Liu *et al.* 2023), and alterations to CTCF binding patterns lead to transcriptional dysregulation (Fang *et al.* 2020).

The CTCF loop landscape is considered to be mostly invariant across similar cell types, and differences on the loop landscape can be linked with changes on the normal cell expression program (Grubert *et al.* 2020). There is evidence that epigenetic disruptions are able to affect CTCF binding and looping (Monteagudo-Sánchez *et al.* 2024), and thus, diseases which present epigenetic alterations as a feature may also feature abnormal CTCF binding and CTCF loop disruption, with Alzheimer’s disease (AD) being an example of possible CTCF disruption (Burton *et al.* 2002).

AD is a neurodegenerative disease and represents the most common form of dementia, affecting over 50 million elderly individuals (“2024 Alzheimer’s disease facts and figures,” 2024). AD is characterized by chronic memory impairment and cognitive deficits related, but not limited to, language, orientation, spatial and temporal awareness, executive capacity, and behavior, a progressive loss of autonomy, dementia, and death (Lane *et al.* 2018). Alzheimer’s is classified into early-onset Alzheimer’s disease (EOAD) and late-onset Alzheimers disease (LOAD) (Atri 2019). EOAD accounts for 1%–2% of all AD cases and it usually presents in patients between 30 and 60 years old (Atri 2019). EOAD is inherited in an autosomal dominant fashion, with a rapid rate of progression, and the most common biomarkers related with EOAD are mutations on the APP, PSEN1, and PSEN2 genes (Atri 2019). In comparison, LOAD accounts for >97% of all AD cases and typically occurs after the age of 65, with many different genetic and environmental factors contributing to the apparition of the disease. In fact, most AD cases are caused by a combination of different risk factors, with age becoming one of the most relevant (Zhao and Huai 2023). AD is characterized histologically by two hallmarks, the intracellular deposition abnormally phosphorylated Tau protein (Maccioni *et al.* 2018), and the extracellular aggregates of Amyloid-beta peptide (AB) plaques (Viola and Klein 2015). AB oligomers may also activate microgial cell signaling cascades that lead to more hyperphosphorilation of Tau protein, which also leads to Tau protein aggregates (Viola and Klein 2015, Maccioni *et al.* 2018). Tau aggregates get released on cell death and also kickstart microglia activation, leading to a cyclic pathological cascade which culminates with cell death and neurodegeneration (Viola and Klein 2015, Maccioni *et al.* 2018). Age-related epigenetic disruptions may be contributing directly to the etiology of AD, since AD and especially LOAD, may be associated with the epigenetic alterations related with aging (López-Otín *et al.* 2023, Yang *et al.* 2023). The CTCF loop landscape in AD is not completely understood, and due to different disruptions in AD epigenetics, abnormal CTCF binding in AD contributing to the etiology of this disease (Patel *et al.* 2023). Currently, the full contribution of CTCF and CTCF loops to AD has not been entirely described, and since epigenetic alterations are a hallmark of AD, those alterations may be contributing to CTCF loop disruption, leading to expression changes on the transcriptional program of the cell (Patel *et al.* 2023).

ChIA-PET is used to detect specific protein-mediated chromatin loops genome-wide at high resolution (Fullwood *et al.* 2009). ChIA-PET requires chromatin cross-linking, proximity ligation of the interacting fragments with linkers, and DNA sequencing of the fragments to estimate the frequency of chromatin interactions (Fullwood *et al.* 2009, Tang *et al.* 2015). Despite recent technical advances, experimental profiling of CTCF-mediated interactions remains difficult and costly (Fullwood *et al.* 2009, Tang *et al.* 2015), therefore, using computational predictions with integrated available datasets can be an interesting alternative to interrogate the CTCF-mediated interactome on a target of interest.

The dynamics of CTCF loops during the development of AD are not completely described, and understanding the dynamics of CTCF loops behind AD may lead to new, undiscovered contributions of CTCF to the etiology of this disease. To understand the dynamics behind CTCF loops, CTCF loop formation can be framed as a classification problem where the presence or absence of certain features contribute to a loop score, such as the conservation of the CTCF binding site, the presence of proteins interacting with CTCF, or the presence of distinct epigenetic marks. Different machine learning approaches have been used in similar biological classification tasks (Greener *et al.* 2022), and to provide new insights about CTCF loop formation in AD we developed a new classifier based on XGBoost (Chen and Guestrin 2016), tested it on different cell lines, and compared it against state-of-the-art CTCF loop predictors (Kai *et al.* 2018, Xu *et al.* 2023). We next predicted loops in control and AD patients, using cell samples from dorsolateral prefrontal cortex. We report a score of loop disruption and report the top disrupted loops on AD. We expect to facilitate *in silico* prediction of CTCF loops, provide new insights about the role of different features on CTCF loop formation, and clarify the role of CTCF looping in AD.

## 2 Methods

### 2.1 Dataset download

We downloaded ChIA-PET data from ENCODE (https://www.encodeproject.org/) for the GM12878 and K562 cell lines and aligned it against the hg19 reference genome using the ChIA-PET2 (Li *et al.* 2017) pipeline which streamlines all the steps required for ChIA-PET data analysis, including trimming, mapping, removal of duplicates, calling of peaks, and calling of chromatin loops. ChIA-PET2 output was transformed to bedpe format, and loops with <2 PETs and FDR >0.05 were removed. We used FIMO (Grant *et al.* 2011) with default values and the MA0139.1 CTCF frequency matrix to identify CTCF binding sites (CTCFBS) on the hg19 reference genome. We also downloaded from the same ENCODE repository, CTCF and RAD21 hg19 ChIP-seq narrowPeak files to define CTCF loops, and h3k4me1, h3k9me3, h3k27me3 and h3k27ac hg19 narrowPeak bed files as extra features for the model for the same cell lines. For GM12878 and K562, the accession codes are: for CTCF ChIP-seq, ENCSR000DZN, ENCSR000EGM, for RAD21 ChIP-seq, ENCSR000EAC, ENCSR000FAD, for h3k4me1 ChIP-seq, ENCSR000AKF, ENCSR000AKS, for h3k9me3 ChIP-seq, ENCSR000AOX, ENCSR000APE, for h3k27me3 ChIP-seq, ENCSR000AKD, ENCSR000EWB and for h3k27ac ChIP-seq, ENCSR000AKC, ENCSR000AKP.

### 2.2 CTCF loop determination

From the bedpe loop file, for each loop, we generated a window of 1000 pb around the start and the end position reported by ChIA-PET2. We removed loops without CTCFBS inside the 1000 pb start and end windows. We only considered loops in the range between 2 kb and over 2 mb, as most of the loops in humans were reported to exist in that pair-bases range (Tang *et al.* 2015). We analyzed the area around three different intervals, the first interval was 1000 pb surrounding the start of the loop, the second interval was 1000 pb surrounding the end of the loop, and the last interval was the area inside the start and the end loop ranges mentioned (interloop).

### 2.3 Feature description and loop prediction

We assigned values to the start and end windows based on the FIMO scores of the CTCF frequency matrix described earlier. If a FIMO predicted CTCFBS was found on either of the start or end intervals, we saved the FIMO predicted CTCFBS score as the start-interval motif score or as the end-interval motif score, respectively. For the rest of the analyzed features, we considered the start-loop interval, the end-loop interval, and the interloop interval windows earlier mentioned. Instead of using the FIMO score, we assigned scores based on the presence or absence of an analyzed feature in the evaluated window. We included CTCF binding, h3k4me1, h3k9me3, h3k27me3, and h3k27ac as analyzed features. After aggregating the loop data, we split the loops into 2/3 and 1/3, using 2/3 of the loops of GM12878 to train the predictor, and tested the predictor with the remaining 1/3 of the loops. We repeated the whole process using the K562 cell line. To confirm if our approach was able to generalize after being trained, we predicted CTCF loops on the K562 cell line using the GM12878 cell line, using the whole GM12878 loops to as input to train the predictor, and the whole K562 loops as a test set.

### 2.4 High-confidence biological loop determination for loop prediction

In humans, the ring-like cohesin complex ring is composed by the SMC1-SMC3 dimer, the kleisin RAD21, and the two SA1 and SA2 cohesin subunits (Gruber *et al.* 2003). We classify a loop as positive when convergently oriented CTCF motifs were occupied by CTCF in any of the start-loop interval, or end-loop interval. We also required colocalization with RAD21 in any of the two CTCF loop anchors, as both interact for the maintenance of chromatin loop domains (Pugacheva *et al.* 2020). We used these loops as high-confidence CTCF loops for training and prediction. Importantly, RAD21 was only used to determine which loops were positive and which loops were negative, and it was excluded as a training feature in the pipeline. We included a diagram of the pipeline as Fig. 5, available as supplementary data at *Bioinformatics Advances* online.

### 2.5 Loop prediction with simulated CTCF binding

Since CTCF ChIP-seq information is not always available, we developed a Random Forests CTCF binding predictor based on different genomic and epigenetic features (Villaman *et al.* 2023), with the objective of using known CTCF-related features on a known cell line to determine CTCF binding on a different cell line without CTCF ChIP-seq data. We evaluated if the predictions made with our predictor were also capable of contributing to the identification of CTCF loops. We generated CTCF binding scores for the GM12878 cell line and compared the loop values with the GM12878 RAD21 loop values, and repeated the same method using GM12878 with simulated values as a training set, and K562 as a test set. We reported precision-recall-area under the curve (PRAUC) and receiver operating characteristic-area under the curve (ROCAUC).

### 2.6 Feature importance in loop prediction

To evaluate feature importance we generated bar plots for each feature used in classification in the GM12878 cell line, K562 cell line, and predicting across cell lines using GM12878 as a training set and K562 as a test set.

### 2.7 Benchmarking with known approaches

We compared our approach with other published approaches (Kai *et al.* 2018, Xu *et al.* 2023), training with GM12878 cell line and predicting on chr2, 20, 21, and 22 of K562 cell line. We reported performance using ROC and PR curves.

### 2.8 AD dataset download

We downloaded information from the ENCODE RUSH AD Project with the objective of having a standard pipeline for data analysis and comparison. We downloaded three GRCh38-aligned control patients without AD, and one patient with AD and cognitive impairment. We downloaded GRCh38 CTCF ChIP-seq data, h3k4me3 ChIP-seq data, h3k27me3 ChIP-seq data, h3k27ac ChIP-seq data, and DNAse-seq data from dorsolateral prefrontal cortex. We also downloaded Hi-C data from the same dataset. We used FIMO with default values and the MA0139.1 CTCF frequency matrix to identify CTCF binding sites (CTCFBS) on the GRCh38 reference genome downloaded from ENCODE, and saved both the FIMO score and the *P* value for posterior use.

### 2.9 AD CTCF binding prediction

From each FIMO predicted AD CTCFBS, we generated matrixes of 500 pb around the AD CTCFBS with 25 pb windows following the same approach described in earlier steps (Villaman *et al.* 2023). We predicted CTCF binding using a Random Forest predictor and compared it with the AD CTCF ChIP-seq dataset. We predicted each patient sample by itself, splitting the sample dataset randomly and using 2/3 of the dataset to train and 1/3 to predict. Then, we trained using two patients as a training set and the remaining one as a test set. To finish, we used the three patients to predict the AD dataset, we used the three patients as a training set and predicted the AD patient as a test set. We reported ROCAUC and PRAUC curves for each.

### 2.10 AD CTCF loop prediction

We used our earlier developed XGBoost CTCF loop predictor to generate prediction scores for every loop on the training set. From the downloaded Hi-C data, we divided loops into positive and negative. Positive loops had two convergent CTCF binding sites on their start and end anchors respectively, between 2 kb and 2 mb pb, with predicted CTCF binding confirmed by ChIP-seq on each. Negative loops were pairs of CTCF binding sites outside the earlier mentioned criteria. We considered an area of 500 pb around the start and end anchors, and the area inside the loop. We considered the presence of a CTCF binding motif, DNA accessibility, h3k4me3, h3k27me3, and h3k27ac as features. We also included the CTCF binding predictions as features for each control and AD sample. We used the three control samples as a training set and the AD sample as a test set. We reported precision, recall, F-score, ROCAUC, and PRAUC (Taha and Hanbury 2015).

### 2.11 Discordant loop determination

We generated scores for every CTCF loop on the control samples under the assumption that CTCF loops in every control sample are relatively constant and related to a normal cell state, and disruptions to loops are related to abnormal cell states such as AD. We used the three control samples as a training set and predicted loop scores on the AD patient, using the same mentioned features for both control and AD dataset. After generating loop scores, we designated discordant loops following any of the two following conditions: (i) Loops on the control dataset with a high predicted score (1) and a low predicted score (<0.0005) on the AD dataset were marked as discordant (looping is lost on the AD dataset), and we define those loops as “lost.” (ii) Loops on the control dataset with a low predicted score 0 and a high predicted score (>0.999) on the AD dataset were marked as discordant (looping is regained on the AD dataset), and we defined those loops as “gained.” We calculated a discordant loop score based on the square of the difference between the loop score obtained on the control dataset and the AD dataset, as shown in the following formula:

To finish, we annotated the loops using the ENCODE GRCh38 gtf, assigning each loop to the single gene with the longest overlap inside the loop, allowing only a single gene for each loop. We ranked genes by the sum of the discordant loop score of loops in which they are within and reported the top 5 genes with the highest discordant loop score.

## 3 Results

### 3.1 Prediction and across-cell prediction

**Figure 1.**
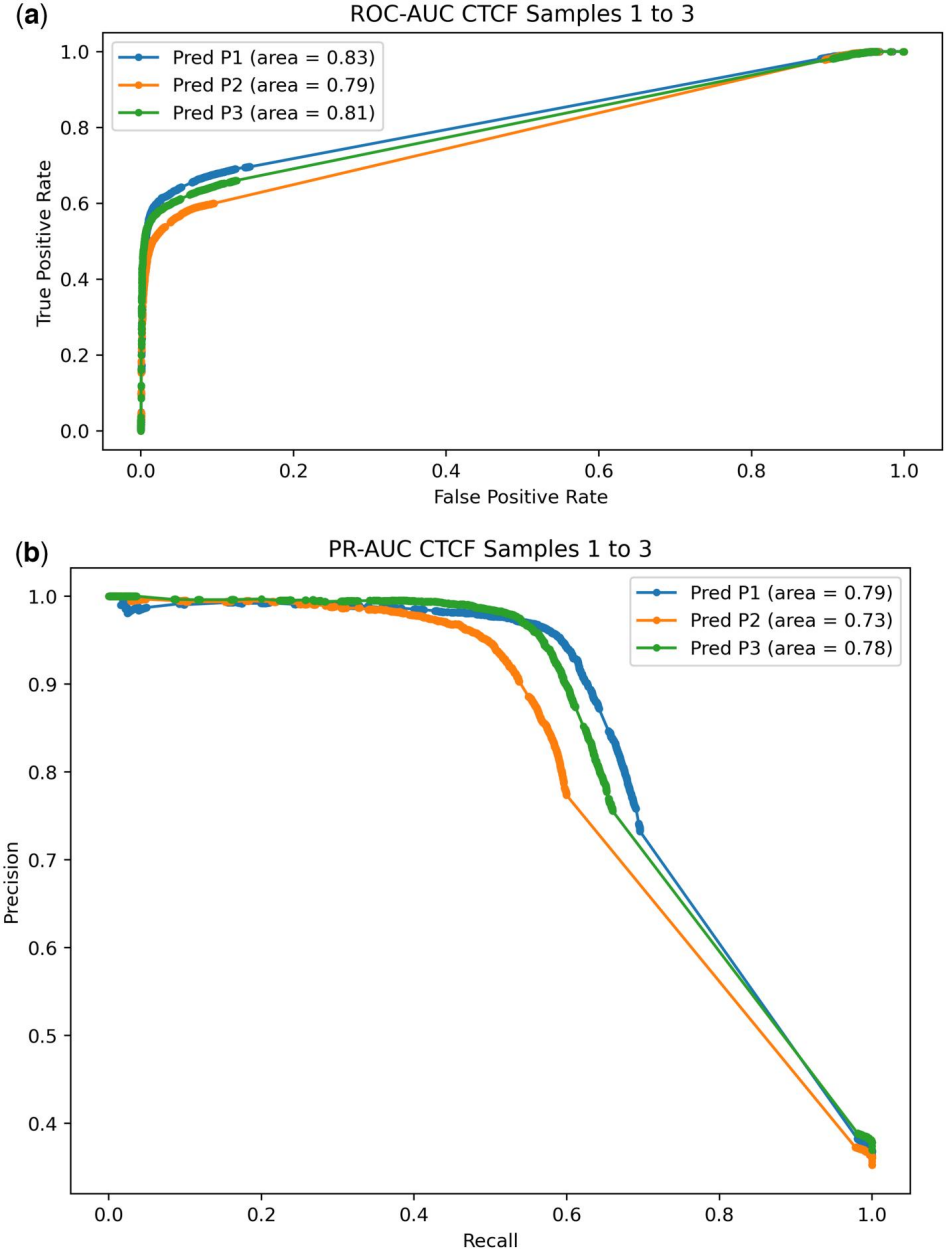
CTCF binding prediction on patients without cognitive impairment, using 2/3rds as a training set and 1/3rd as a testing set, using each patient to predict itself. (A) ROC-AUC. (B) PR-AUC.

We selected 25 000 and 19 070 positive loop examples for GM12878 and K562, defining a positive loop with 2 bound CTCF sites on its boundary and RAD21 on any of the three reported loop windows (start, end, and intraloop). Negative examples were selected by picking two unbound CTCFBS without RAD21 on any of the loop windows mentioned. We tested GM12878 against itself, and K562 against itself as a first approach, by randomly splitting the dataset using 2/3 to train and 1/3 to test. To test the performance of the predictor on different cell types, we used GM12878 as a training set and K562 as a test set. We reported precision, recall and f-score of over 0.83 in all of the predicted cell lines, however, prediction across cell lines has lower scores than training and prediction with the same cell line (Table 1 and Fig. 1A and B, available as supplementary data at *Bioinformatics Advances* online).

**Table 1.** Precision, recall, and F-score after predicting CTCF binding.^a^

Sample	Precision	Recall	F-score
Pred P1 Sample 1	0.92	0.62	0.74
Pred P2 Sample 2	0.93	0.58	0.72
Pred P3 Sample 3	0.91	0.54	0.67
Pred P1 Cross 1	0.93	0.58	0.71
Pred P2 Cross 2	0.91	0.53	0.67
Pred P3 Cross 3	0.93	0.62	0.74
AD Predict	0.75	0.59	0.66

a Sample 1, 2, and 3 uses information from said sample, splits randomly the CTCFBS on the sample using 2/3rds as a training set and 1/3rd as a test set. Cross 1, 2, and 3 represents using two samples as a training set and predicting on the remaining one as a test set. AD Predict uses the three control patients to predict a patient with AD and cognitive impairment.

### 3.2 Prediction based on CTCFBS prediction

**Figure 2.**
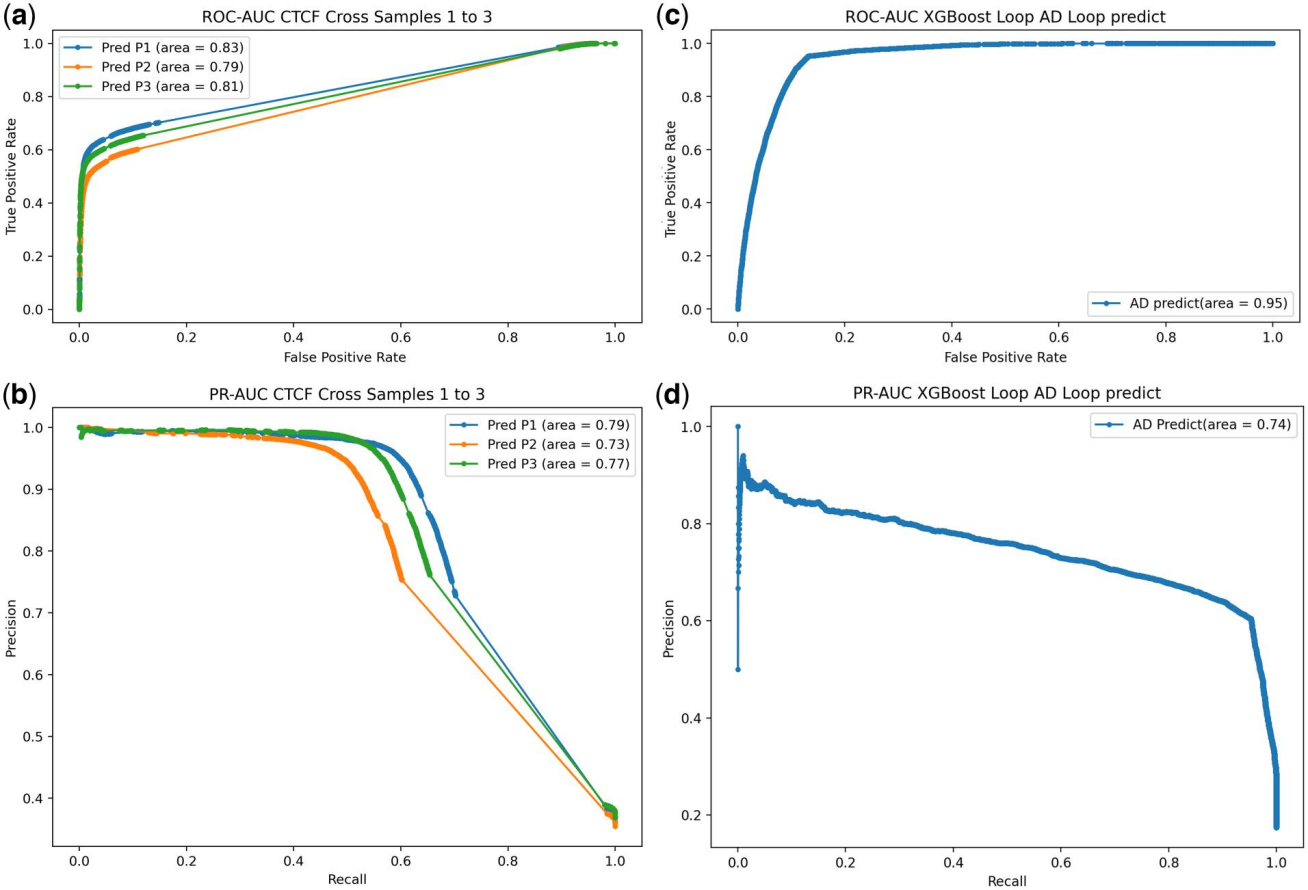
CTCF binding prediction on patients without cognitive impairment and patients with AD and cognitive impairment. (A) ROC-AUC predicting CTCF binding on three controls using two controls as a training set and the remaining one as a test set. (B) PR-AUC of the same controls. (C) ROC-AUC, using three control samples to predict the AD sample. (D) PR-AUC using three control samples to predict the AD sample.

CTCF ChIP-seq experiments are not always available, and to deal with this problem we developed a CTCF binding predictor based on different genetic and epigenetic features (Villaman *et al.* 2023). Since CTCF binding is a very important feature on the determination of CTCF loops, we wanted to test if the results from our predictor were able to be used as features for CTCF loop prediction. We trained the predictor using a similar protocol to the one mentioned earlier, in this case we employed the GM12878 cell line, considering DNA accessibility, h3k4me1, h3k9me3, h3k27me3 and h3k27ac histone marks, DNA methylation, and FIMO motif score, and used the score of the predictor instead of the experimental CTCF ChIP-seq data. We used the same approach as the first prediction, we randomly split the dataset using 2/3 to train and 1/3 to test. We also used the scores for testing across cells, using the predicted GM12878 CTCF binding scores as the training set and K562 CTCF ChIP-seq data as test set. We reported precision, recall and f-score of over 0.86, with prediction across cells having a lower score than training and prediction with the same cell line (Table 2 and Fig. 2A and B, available as supplementary data at *Bioinformatics Advances* online).

**Table 2.** Top five most disrupted loops for gained and lost loop instances.^a^

chr	startloop	endloop	Predicted score	Discordant score	Gene name
chr10	124025000	124095000	8.93E-05	0.999821	CHST15
chr17	59440000	59570000	0.000123	0.999752	DHX40
chr11	70467000	70756000	0.000143	0.999713	SHANK2
chr12	40970000	41070000	0.000174	0.999651	CNTN1
chr2	74915000	75405000	0.000182	0.999635	POLE4
chr7	71750000	72640000	0.999581	0.999163	CALN1
chr7	71750000	72640000	0.999581	0.999163	TYW1B
chr6	33410000	33580000	0.999782	0.999564	SYNGAP1
chr6	33410000	33580000	0.999782	0.999564	GGNBP1
chr8	130290000	130680000	0.999925	0.999851	ASAP1

a Top five are lost loops, Bottom five are gained loops.

### 3.3 Feature importance determination

To gain insights about the contribution of the different evaluated features into the prediction, we extracted and reported the feature importance of every feature used in classification. The most important feature was the FIMO score at the start of the loop, followed by the presence of bound CTCF at the end of the loop. This is expected and it accounts for how the CTCF loop is being formed; if the start of the loop is conserved or bound, we can expect to find a loop if the end of the loop is also conserved or bound. Following these observations, after binding on the end of the loop the next important feature is FIMO score on the end, and then CTCF binding inside the loop, which may consider unaccounted CTCF binding nearby the start or the end of the loop. After those features, the following features were the loop distance and the histone marks, on any of the reported starts and ends of loops (Fig. 3A–C, available as supplementary data at *Bioinformatics Advances* online).

### 3.4 Comparison with other methods

We compared the performance of this method against two other state-of-the-art methods (Kai *et al.* 2018, Xu *et al.* 2023). We picked Lollipop (Kai *et al.* 2018) due to being one of the legacy CTCF loops predictor, and we used LoopAnchor (Xu *et al.* 2023) due to being one of the latest published CTCF loops predictor by the time of writing. We trained each predictor with the default features for each, tested training with GM12878 using default values and features for each method, and used chromosomes 2, 20, 21, and 22 of K562 cell line as a test set. We report ROCAUC and PRAUC of each predictor, finding that our predictor has a slightly lower ROCAUC than the other predictors, and a better performance in terms of PRAUC than Lollipop, but slightly worse than LoopAnchor (Fig. 4A and B, available as supplementary data at Bioinformatics Advances online).

### 3.5 AD CTCF binding prediction

We used FIMO to predict CTCF binding sites on the GRCh38 reference genome, and used the FIMO scores with four other different features (CTCF binding motif, DNA accessibility, h3k4me3, h3k27me3, and h3k27ac) to train a Random Forest predictor for CTCF binding on three different control AD patients, using CTCF ChIP-seq information as gold standard; 52 435 CTCF binding sites were predicted by FIMO. We first analyzed each patient by itself, splitting the whole dataset into 2/3 used for training and 1/3 used for testing and reported precision, recall, and F-score, and plotted ROCAUC and PRAUC (Table 1, Fig. 1A and B). After predicting each patient by itself, we used two patients to predict the third one, to increase the number of available examples and to confirm if the predictor was able to generalize. We reported precision, recall, and F-score, and plotted ROCAUC and PRAUC (Table 1, Fig. 2A and B). For the AD dataset we trained the model, used the three patients datasets and used it to predict the patient with cognitive impairment and AD, and reported performance afterwards (Table 1, Fig. 2C and D).

### 3.6 CTCF loop prediction

For CTCF loop prediction we downloaded Hi-C loop data from ENCODE and assigned positive and negative loops considering positive loops as loops with convergent, CTCF-bound CTCF binding sites with a distance between 2 kb and 2mb, considering earlier mentioned features. We trained a XGBoost loop predictor and generated predictions for each loop using controls as a training set and AD patient as a test set. To finish, we generated scores for loops on the AD sample and saved them for posterior use.

### 3.7 Discordant loop determination

We compared the obtained loop scores between the control sample and the AD sample, and divided the scores between gained loops (loops with a low score on the control sample and high score on the AD sample) and lost loops (loops with a high score on the control sample and low score on the AD sample), and ranked them based on their discordant loop score (Table 2). The top predicted lost loops involved the genes CHST15, DHX40, SHANK2, CNTN1, and POLE4, and the top predicted gained loops contained SYNGAP1, GGNBP1, TYW1B, CALN1, and ASAP1. According to bibliography, four of the five top lost loops have been linked to AD with supported evidence, suggesting a possible link between loop disruption and AD (Table 3).

**Table 3.** Genes in disrupted loops and their link with AD.

Gene	AD link	Citation	DOI
CHST15	Neuropathological AD-related gene	Silva *et al.* (2012)	10.1371/journal.pone.0048751
DHX40	Age-dependent differentially methylated	Pellegrini *et al.* (2021)	10.3389/fnagi.2021.639428
SHANK2	Decreased in AD	Patel *et al.* (2023)	10.1016/j.nbd.2023.106192
CNTN1	Aggravates neuroinflammation in AD	Li *et al.* (2023)	10.14336/AD.2023.0228
POLE4	Reduced CTCF binding in AD	Patel *et al.* (2023)	10.1016/j.nbd.2023.106192
CALN1	Differentially expressed in AD	Li and De Muynck (2021)	10.1016/j.bbih.2021.100227
TYW1B	Detected in AD neural networks	Ghose *et al.* (2024)	10.1093/bib/bbae704
SYNGAP1	Risk factor in GWAS AD	Mez *et al.* (2017)	10.1016/j.jalz.2016.09.002
GGNBP1	Gametogenetin-binding protein 1, unknown function, no known AD link.	–	–
ASAP1	Genetic modulator for Tau pathologies	Xu *et al.* (2024)	10.1007/s00401-024-02703-3

## 4 Discussion

CTCF is related with multiple functions inside the cell and determining the full extension of its phenotypic and mechanistic roles remains to be done. Over 60% of the CTCF binding sites are conserved across different cell lines (Chen *et al.* 2012), and Rao *et al.* (2014) reported from 55% to a 75% of shared conserved peaks in HiC experiments across them. Under this context, FIMO score can be considered as a proxy of CTCF binding site conservation, and FIMO score is consistently one of the most important features in classification reported by our predictor. Both FIMO score and CTCF binding were reported as the most important features in CTCF loop classification, and due to the conservation of CTCF binding sites between different tissues and cell lines, the detection of conserved CTCF loops is possible using only the CTCF motif scores with our approach. Other methods to predict CTCF loops exist, however, they differ in both methods and performance (Kai *et al.* 2018, Zhang *et al.* 2018). One advantage of this method is prediction across cell lines, however, a performance reduction in our method is to be expected due to differences between each particular cell CTCF landscape. Nevertheless, other approaches have focused on the importance of the motif sequence and have predicted loops only training with the CTCF binding sequences (Zhang *et al.* 2018) and our results confirm the importance of these observations.

Cell-specific CTCF loops with lower conservation scores may require different considerations than conserved CTCF loops, for example, less conserved loops may determine specific cell processes related directly with lineage specification (Liu *et al.* 2023), and training on different cell lines may not be able to provide proper examples for training, interfering with the identification of loops across cell lines. Since CTCF cooperates with lineage-specific pioneer transcription factors (TFs), such as MyoD, FOXA, and PU.110, including expression data of these factors may improve cell-specific loop resolution. We considered only the presence of features inside the loop, or around the start or end of the loop, since we deemed the presence of a feature to be more relevant than its exact location on the sequence. However, we can increase the resolution by creating bins inside the analyzed regions of the CTCF loop to improve performance, especially if features related to opposite biological phenomena occupy the same region, such as DNA methylation and DNA expression, or h3k27ac and h3k27me3.

We reported relatively good performance using *in silico* predicted CTCF binding scores using two different cell lines. The two cell lines were selected according to the assays available at the time of the study. K562 is a widely used human leukemia cell line with an extensive genomic and epigenomic characterization (Zhou *et al.* 2019), while GM12878 is an EBV-transformed human B-lymphoblastoid cell line (Lin *et al.* 2022). It is well known that bypassing cellular senescence, either due to the tumoral cellular origin or via the immortalization process of primary cells, required additional mutation and/or epigenetic alterations. These mechanisms contribute to both inter- and intra-cell line heterogeneity and may have an influence on results outcomes (Zhu *et al.* 2023). Nonetheless, our work proposes a useful model to predict CTCF loops, which will be further strengthened as more human cell lines are characterized by multiomics profiling assays.

We considered overfitting as a possible issue, as we have evaluated the use of GM12878 multiple times during the development of this work. However, in earlier work by the same authors (Villaman *et al.* 2023), and other methods of TF binding prediction such as msCentipede (Raj *et al.* 2015) and Catchitt (Keilwagen *et al.* 2019) also report improved results on GM12878 when compared to other cell lines from the ENCODE dataset. We believe that the results are not related to overfitting, and they are related with better quality of the hg19 GM12878 dataset when compared to the rest of the ENCODE datasets.

Testing across multiple cell lines would improve the number of available examples, improving performance on shared loops, however, an impaired performance could be expected on cell-specific loops. Including additional features such as methylation, gene expression, or DNA accessibility would contribute to identify cell-specific loops if the conservation of their CTCF binding sites is low, nonetheless loops with weaker CTCF binding would most likely be exclusive to the cell and related with its own transcriptional landscape, making possible that the inclusion of extra features does not improve the performance of the predictor. Nevertheless, the relationship between performance improvement versus performance impairment remains to be studied. We were able to predict with over 0.9 F-score on the analyzed cell lines, with the worst performance obtaining when predicting training across cell lines, highlighting the idea that conserved loops share similar features while cell-specific loops present singular features. We plan to expand the array of both features and cell lines included on CTCF loop prediction to improve prediction performance, and at the same time to provide new insights about the role of CTCF loops across the myriad of existing human cell types.

Importantly, our results suggest the existence of two different kind of CTCF binding sites, conserved ones shared across cells, and cell-exclusive CTCF binding sites. In fact, there is evidence sustaining that conserved CTCF sites have a structural function while non-conserved ones have a regulatory function (Plasschaert *et al.* 2014, Marina-Zárate *et al.* 2023). Since there is a direct relation between CTCF binding site conservation and loop presence (Zhang *et al.* 2018), loops can be divided between shared loops with high CTCF binding site conservation between cell lines, and less conserved cell-specific loops. The samples analyzed from dorsolateral prefrontal cortex were sequenced from a combination of many different cell types on different states, in both the controls and the AD and cognitive impairment samples, and it is plausible that our predictor was capable of resolving shared CTCF binding sites but not cell-exclusive CTCF binding sites. This also supports the idea that there are also two kinds of CTCF loops, ones formed between conserved CTCF binding sites and cell-exclusive loops formed between less conserved CTCF binding sites. This idea is confirmed by many different authors (Martin *et al.* 2011, Wang *et al.* 2021, Xu *et al.* 2023). If the predictor is not able to properly identify CTCF binding, it would also not be able to properly resolve the cell-exclusive CTCF loops since they are dependent on the CTCF binding score feature. Another factor is the idea that changes in control patients and AD samples are related with the cell composition and cell death inherent with AD (Johnson *et al.* 2020), however, since the CTCF loop landscape is mostly invariant, it is possible to suggest that the changes in CTCF loops, especially those with a high CTCF binding score, account directly for AD-related abnormalities and are shared across the analyzed tissue cell types.

Another issue to consider is the fact that changes in CTCF binding and looping may not affect gene expression directly, however, loss of CTCF binding may prevent the formation of CTCF loops in response to cellular events, affecting the ability of the cell to maintain homeostasis (Kaushal *et al.* 2021, Xu *et al.* 2021), this suggesting that changes to CTCF in AD may be a cumulative trait during the development of this disease. A proper progression of CTCF loop disruption in AD cannot be established due to the lack of available data, but we expect that as more AD datasets become available, more information would be available about AD and CTCF loop during the development of the disease.

There are reports of lower CTCF binding in AD samples, suggesting altered methylation as a possible mechanism of CTCF binding disruption (Patel *et al.* 2023). Conserved CTCF binding sites are shared across cell lines, and impaired CTCF binding on conserved CTCF sites may be a hallmark of this disease. However, if the loss of CTCF binding is random or gene-specific remains to be determined. Abnormal DNA methylation is a hallmark linking CTCF and different kinds of cancer (Damaschke *et al.* 2020), this connection can be explained by the abrogation of CTCF binding by methylation and the development of cancer-specific expression landscapes leading to cancer-specific hypermethylation (Damaschke *et al.* 2020), but if the loss of CTCF in AD leads to AD-specific methylation and gene expression is not yet verified.

Importantly, we found discordant loops and divided them in two groups, loops gained on AD and loops lost in AD. We suggest that loops gained in AD are representative of the different cell composition during the development of AD while comparing AD and control samples, and loops lost on AD are directly related with impaired CTCF binding shared across cells in the dorsolateral prefrontal cortex. CTCF binds to unmethylated DNA sequences (Holwerda and De Laat 2013, Prickett *et al.* 2013, Maurano *et al.* 2015) and it can bind to DNA damage sites and activate a cascade reaction resulting in DNMT1 inactivation and DNA demethylation (Zampieri *et al.* 2012). Moreover, CTCF is also related to patterns of histone modifications and is especially required to implement both H3K27ac and H3K27me3 (Wang *et al.* 2021). While their defects may rewire genome-wide chromatin accessibility and have serious implications on the normal cell expression program (Xu *et al.* 2021). Thus suggesting that the decline in CTCF binding with age may be an initial mechanism in AD pathogenesis (Wang *et al.* 2020, Hou *et al.* 2021). One of our main issues was data availability, as the Hi-C experiments required to confirm CTCF loops are not readily available for the conditions we wanted to evaluate, and we have not evaluated our approach in other datasets again due to the earlier lack of data mentioned. While it may not be possible to extend the results to the whole spectrum of AD etiology due to an inherent lack of robustness, we can also acknowledge the fact that the genes involved in the loops predicted as lost have also been experimentally described as relevant in AD, such as SHANK2 (Patel *et al.* 2023) and POLE4 (Ferrer *et al.* 2021, Patel *et al.* 2023). Patel and collaborators also analyzed the RUSH ENCODE dataset, however, while there is an overlap in controls, there is no overlap in AD cases, and while our results could be describing an isolated case, we believe that the similarity in results highlights and underlying mechanism and not a singular phenomenon. Nevertheless, caution should be advised while interpreting these results, and we expect to expand on them on further analyses.

We report CTCF loop loss on genes directly related with AD on AD sample dorsolateral prefrontal cortex, however, we lack enough information to suggest the mechanism behind it. There is also evidence of interactions between APOE and CTCF and it is possible for CTCF to make protein complexes directly with AB peptides or Tau hindering its ability to bind DNA (Del Moral-Morales *et al.* 2023), however, more studies on the topic are required to confirm the existence of either a pattern or a mechanism confirming the role of CTCF and CTCF looping in AD. Finally, our evidence shows concrete links between epigenetics, CTCF, and the etiology of LOAD, and the exploration of those links may provide new clues about development of LOAD and possible therapeutic targets to this disease in the future.

## 5 Conclusion

We developed a CTCF loop predictor that can use CTCF binding predictions with similar performance to other state-of-the-art predictors and used it to gain new insights on AD. We identified that CTCF binding site conservation and CTCF binding are the most important features in loop classification, consistent with observations on different cell lines. We identified that conserved CTCF binding sites and conserved CTCF binding are related with CTCF loops, and CTCF loops with weaker conservation scores and CTCF binding may be related with cell-specific CTCF binding, requiring the inclusion of more features to be properly resolved. We predicted CTCF binding and CTCF loops in three control samples and an AD sample using genetic and epigenetic features. We reported loop disruption when comparing the control and the AD sample and found that the top disrupted loops were genes that were reported as AD-related in bibliography. We expect to expand on both the amount of analyzed cell lines and features included in the nearby future to provide new knowledge about CTCF function across different cell lines. Importantly, with the currently available information it is not possible to elucidate a clear biological mechanism besides correlation, and more studies are required to confirm the extent of disruption of CTCF binding and CTCF loops in AD. Nevertheless, our observations support the fact that there is a link between CTCF and the etiology of AD.

## Supplementary Material

vbaf268_Supplementary_Data

## Data Availability

The data employed in this article is available in ENCODE data repository (https://www.encodeproject.org/), accession IDs ENCSR000DZN, ENCSR000EGM, ENCSR000EAC, ENCSR000FAD, ENCSR000AKF, ENCSR000AKS, ENCSR000AOX, ENCSR000APE, ENCSR000AKD, ENCSR000EWB, ENCSR000AKC, and ENCSR000AKP. All scripts employed can be found at https://github.com/networkbiolab/jalpy.
