# Supplementary material for: Gaining insights into Alzheimer’s disease by predicting chromatin spatial organization
Source: Bioinform Adv. 2025 Oct 25;5(1):vbaf268. doi: 10.1093/bioadv/vbaf268 (PMC12627407; doi:10.1093/bioadv/vbaf268)
Supplement: vbaf268_Supplementary_Data [file vbaf268_supplementary_data.docx]

| **type** | **precision** | **recall** | **f1-score** |
| --- | --- | --- | --- |
| gm12878 | 0.96 | 0.99 | 0.97 |
| k562 | 0.88 | 0.94 | 0.91 |
| gm-to-k | 0.98 | 0.83 | 0.90 |


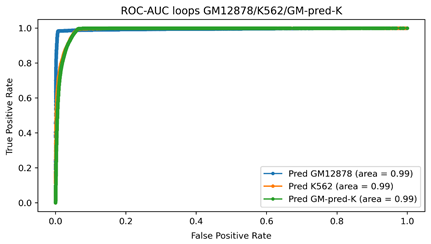
Supplementary
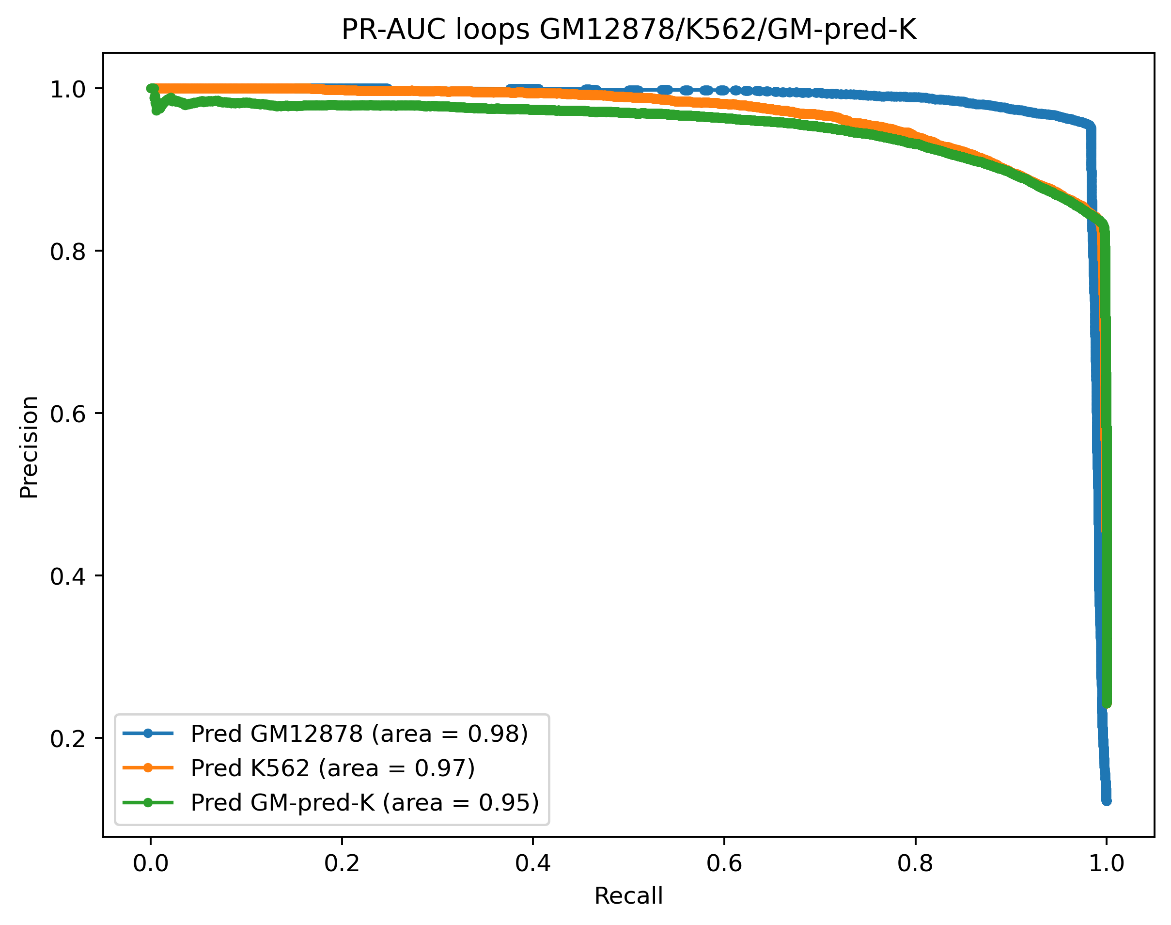
Table 1: Precision, Recall and F-Score according to the predicted cell line.

B)

A)

Supplementary Figure 1: A) ROC-AUC using GM12878, K562 and GM12878 to predict K562 loops. B) PR-AUC using GM12878, K562, and GM12878 to predict K562 loops.

| **Type** | **precision** | **recall** | **f1-score** |
| --- | --- | --- | --- |
| gm12878 | 0.93 | 0.98 | 0.96 |
| gm-to-k | 0.76 | 0.99 | 0.86 |


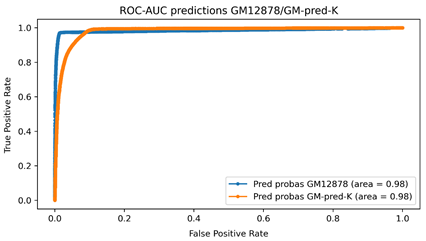

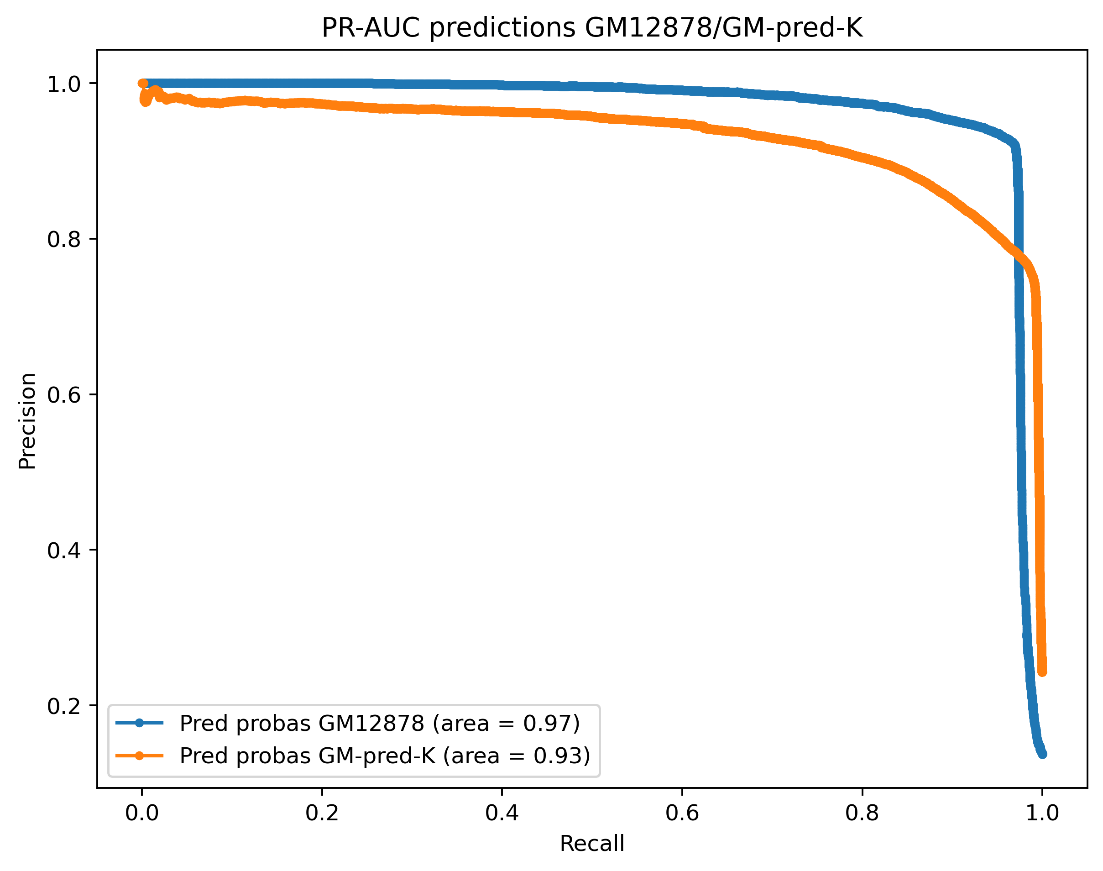
Supplementary Table 2: Precision, Recall and F-Score using predicted CTCF scores for each cell line.

B)

A)

Supplementary Figure 2: A) ROC-AUC using GM12878 predictions and GM12878 predictions to predict K562 loops. B) PR-AUC using GM12878 predictions and GM12878 predictions to predict K562 loops.


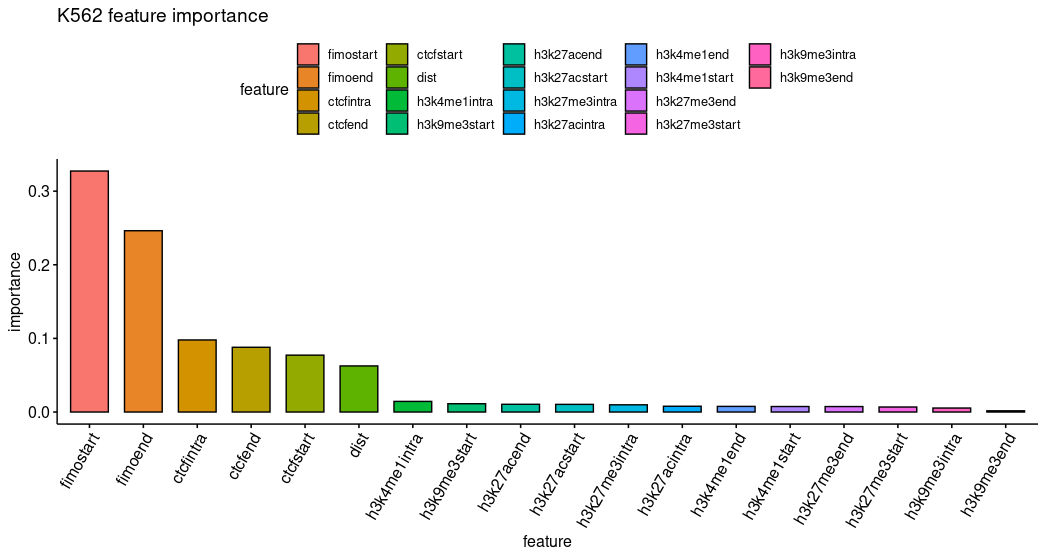

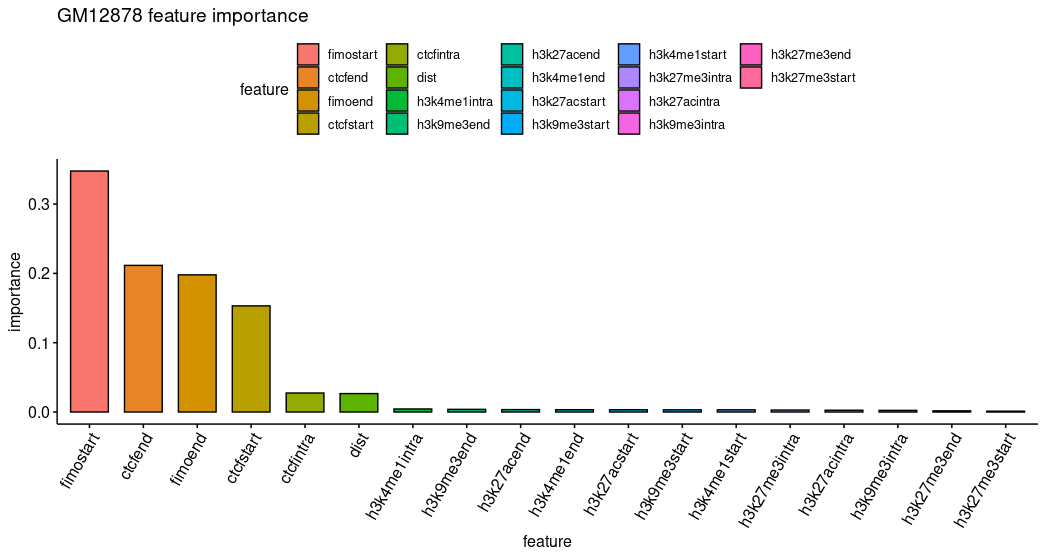

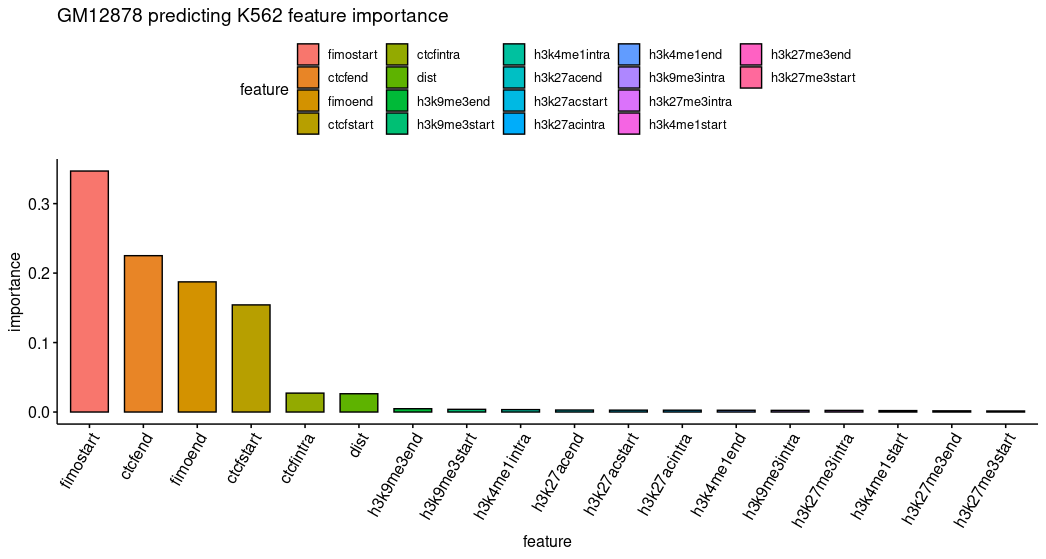
Supplementary Figure 3: A) Feature importance predicting loops in GM12878 cell line. B) Feature importance predicting loops in K562 cell line. C) Feature importance using GM12878 to predict K562 loops. FIMO: FIMO score. CTCF: CTCF binding. Dist: Loop distance. Histone: Histone mark. Start/End/Intra: Localization on the start/end/intra loop space.

C)

B)

A)


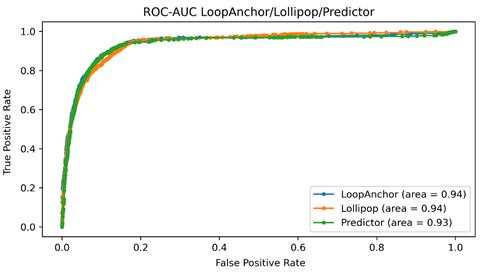


B)

A)

Supplementary
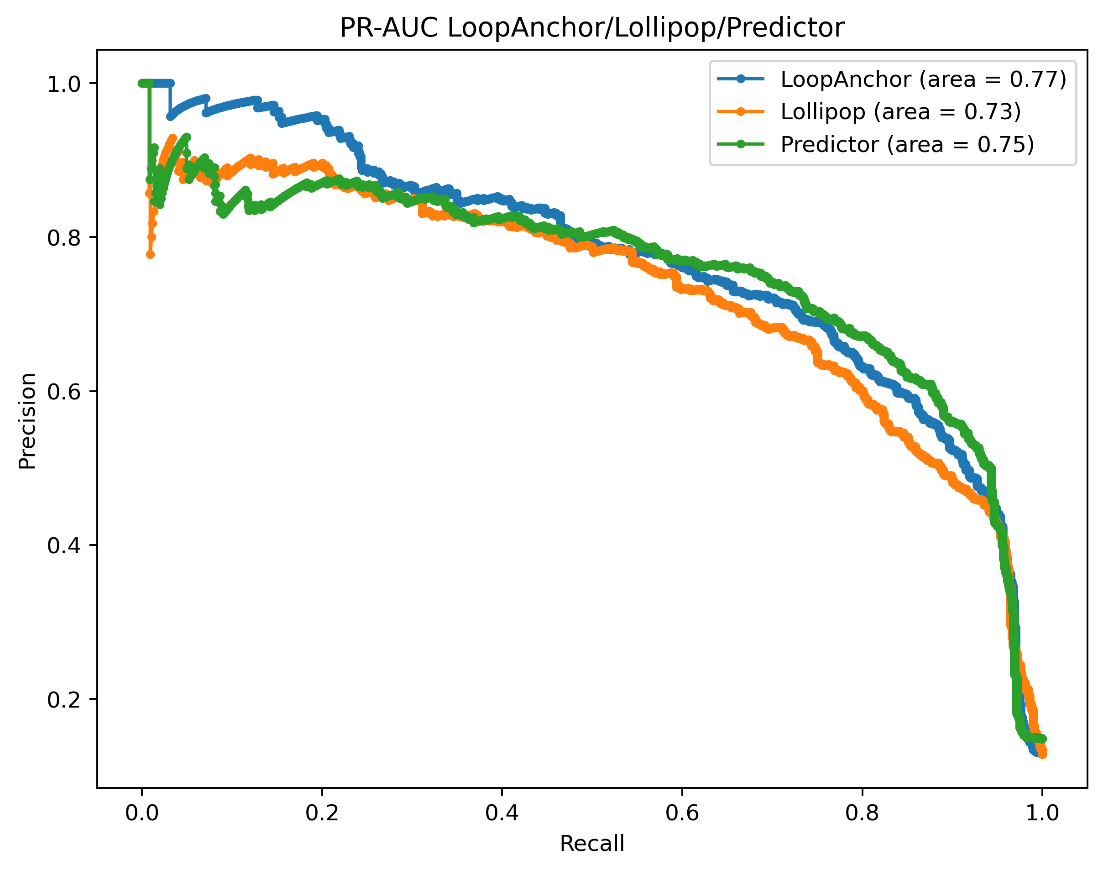
Figure 4: A) ROC-AUC using LoopAnchor, Lollipop and XGB predictions training with GM12878 and predicting against chr2, chr20, chr21 and chr22 of K562. B) PR-AUC using using LoopAnchor, Lollipop and XGB predictions training with GM12878 and predicting against chr2, chr20, chr21 and chr22 of K562.

B)


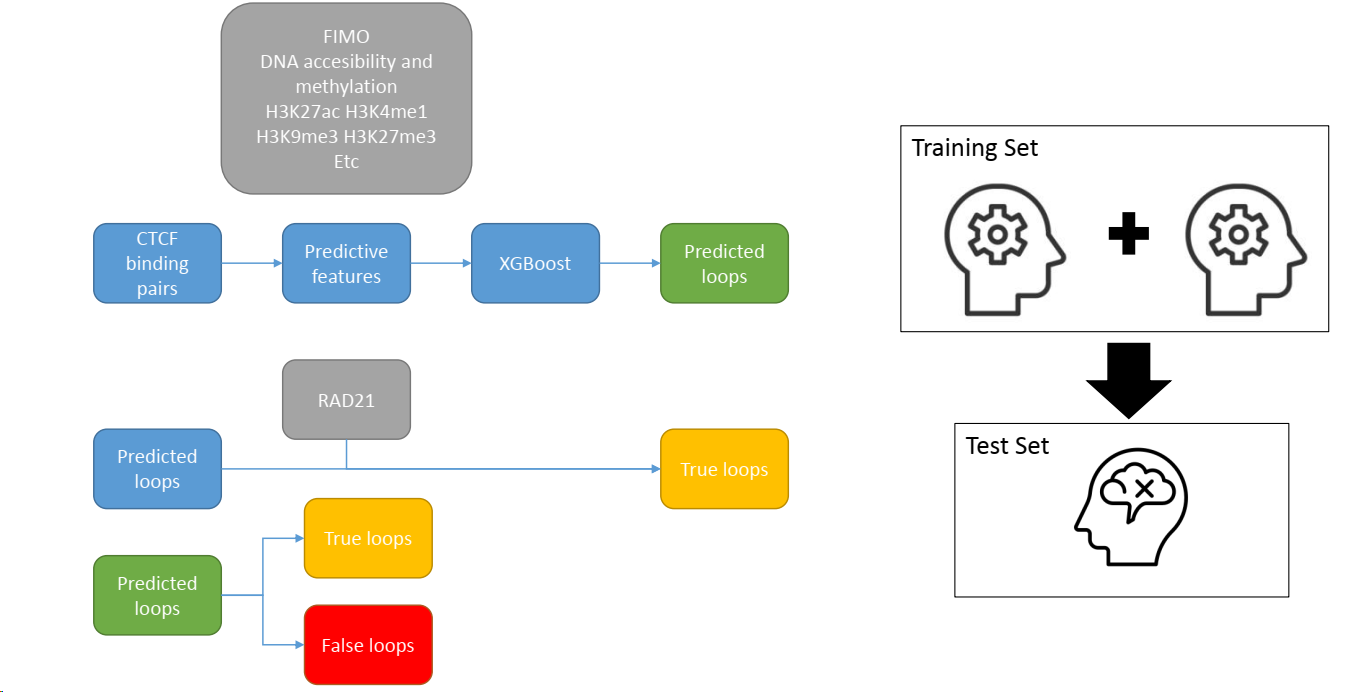


Supplementary Figure 5: Fluxogram explaining the idea behind the predictor.
